# Source-Specific Nitrogen Inputs Are Associated with Pathway Partitioning Between Denitrification and DNRA in River Water

**DOI:** 10.3390/biology15100741

**Published:** 2026-05-08

**Authors:** Qianhang Sun, Jiangnan Li, Guohui Xu, Chunhe Zhou, Kun Lei, Weijun Jiang

**Affiliations:** 1 College of Environmental Science and Engineering, Ocean University of China, Qingdao 266000, China; 2 Lishui Ecological and Environmental Monitoring Center of Zhejiang Province, Lishui 323000, China; 3 Key Laboratory of Estuarine and Coastal Environment, Ministry of Ecology and Environment, Chinese Research Academy of Environment Sciences, Beijing 100012, China

**Keywords:** nitrogen source, river water, DNRA, denitrification, nitrogen retention

## Abstract

Understanding how external nitrogen sources regulate nitrogen fate in river water is critical for improving nitrogen removal and reducing greenhouse-gas risk. Here, short-term microcosm incubations were conducted using source water as the background matrix and seven representative source inputs. By integrating hydrochemical analyses, bacterial community profiling, metagenomics, RT-qPCR, and process-rate measurements, we evaluated source-dependent shifts in nitrogen-cycling pathways. Manure-related inputs generated the highest organic and nitrogen loading, suppressed nitrification, enhanced *nrfA (cytochrome c nitrite reductase)* abundance and transcription, and promoted DNRA, indicating a shift toward nitrogen retention via ammonium regeneration. In contrast, sewage-related inputs maintained relatively high NO_3_^−^ availability, elevated *nirS (cytochrome cd1 nitrite reductase)* and *nosZ (nitrous oxide reductase)* expression, and enhanced denitrification, but also increased N_2_O production. Metagenomic, transcriptional, and rate-based evidence consistently identified 12 h as a critical window for source-dependent pathway redistribution, highlighting the importance of short-term monitoring for detecting rapid nitrogen-cycle responses following pollution inputs. These findings support source-oriented nitrogen management that considers both nitrogen loading and hydrochemical controls on nitrate fate.

## Simple Summary

Different nitrogen sources do not affect river water in the same way. This study examined how manure, sewage, and other common inputs change the path of nitrogen after it enters river water. We found that manure-related inputs created carbon-rich, oxygen-poor conditions that shifted nitrogen toward ammonium, a form that remains in the water and can continue to support algal growth. In contrast, sewage-related inputs more often promoted conversion of nitrogen to gas, which can help remove nitrogen from water, but they also increased the risk of producing a heat-trapping gas. These changes happened quickly, with a clear shift appearing within about twelve hours after pollution entered the water. The results show that improving river water quality requires not only reducing nitrogen input, but also controlling pollution source types and the water conditions that determine whether nitrogen is removed or retained.

## 1. Introduction

Nitrogen pollution remains a major threat to riverine water quality because it not only increases nutrient concentrations, but also alters the capacity of aquatic systems to remove, recycle, or retain reactive nitrogen [1,2]. In human-impacted rivers, external nitrogen loading is often accompanied by shifts in dissolved oxygen, organic matter supply, and other geochemical conditions, such that different source inputs may be associated with contrasting nitrogen fates, even under broadly comparable nitrogen conditions [2,3]. Therefore, the environmental consequence of nitrogen pollution depends not only on loading magnitude, but also on how nitrate is partitioned among competing microbial pathways after entering receiving waters [1,3].

Reactive nitrogen can be transformed through nitrification, denitrification, dissimilatory nitrate reduction to ammonium (DNRA), and anaerobic ammonium oxidation (anammox) [3,4,5]. These pathways differ fundamentally in their implications for water quality: denitrification and anammox contribute to permanent nitrogen removal, whereas DNRA retains bioavailable nitrogen by reducing nitrate to ammonium and may thereby prolong eutrophication pressure [1,6,7]. In addition, incomplete denitrification can increase N_2_O production, linking river nitrogen pollution to greenhouse-gas emissions [8]. Clarifying how external nitrogen sources redistribute nitrate among removal, retention, and emission pathways is therefore essential for understanding the self-purification potential and environmental risks of polluted rivers [1,3].

Pathway partitioning is highly sensitive to local water chemistry. Oxygen availability regulates the balance between oxidative and reductive transformations, whereas organic carbon availability strongly affects nitrate-reducing processes and the competition between denitrification and DNRA [2,6,9]. These controls are especially relevant in human-impacted rivers because pollution sources differ markedly in chemical composition. For example, manure-related inputs are often characterized by higher ammonium and labile organic carbon, whereas treated effluents are more frequently associated with nitrate enrichment and lower biodegradable carbon, potentially driving distinct nitrogen-cycling trajectories [2,7,8].

Microorganisms directly mediate these transformations, and shifts in microbial community composition and functional potential provide the mechanistic basis for source-dependent nitrogen fate [3]. Previous studies have shown that nutrient enrichment can alter the abundance and activity of nitrifiers, denitrifiers, and DNRA-associated microorganisms, thereby changing the relative importance of individual nitrogen pathways [7,9,10]. However, comparatively few studies have linked source-specific water chemistry, microbial community restructuring, nitrogen-cycling gene dynamics, and measured process rates within a unified framework, particularly for the short-term responses following pollution inputs [8,10]. This knowledge gap limits our ability to explain why some external inputs enhance nitrogen removal, whereas others favor nitrogen retention or increase N_2_O risk.

In this study, we established short-term microcosm incubations using source water as the background reference and multiple representative nitrogen-source inputs. We integrated analyses of water chemistry, carbon-to-nitrogen conditions, and metal ion backgrounds with microbial community profiling, metagenomic annotation of nitrogen-cycling modules, transcriptional quantification of key functional genes, and direct measurements of DNRA, denitrification, and N_2_O production rates. We hypothesized that source-specific inputs would create distinct nitrogen-cycling trajectories by altering substrate supply and redox conditions. Specifically, manure-related inputs with high organic loading and elevated ammonium concentrations were expected to suppress nitrification and shift nitrate reduction toward DNRA, whereas sewage- and effluent-related inputs were expected to favor denitrification-related pathways and increase the potential for N_2_O production under nitrate-rich conditions. By linking environmental gradients, microbial responses, functional gene dynamics, and process rates, this study aims to clarify how external nitrogen sources regulate nitrogen fate in receiving waters and to provide a process-based basis for source-oriented river water quality management.

## 2. Materials and Methods

### 2.1. Microcosm Setup and Incubation

To assess the short-term effects of different external nitrogen sources on nitrogen-cycling pathways in river water, source water (YS) was used as the background matrix to establish a series of source-input microcosms, and its basic physicochemical and microbial characteristics are summarized in Table S1. Eight treatments were included: source water control (YS), wastewater treatment plant effluent (WS), rural domestic sewage (NW), agricultural tailwater (ST), aquaculture effluent (YU), soil runoff (TR), chicken manure input (JF), and pig manure input (ZF). Each treatment was prepared in triplicate using sterile glass bottles.

Fresh source water and endmember samples were collected from the corresponding sites and transported to the laboratory under cooled conditions on the same day. Microcosms were established by mixing each source input with source water at a fixed volumetric ratio. Control bottles contained source water only. All bottles were incubated in the dark at 20 ± 1 °C without shaking to simulate short-term conditions following source input into receiving river water. Each treatment was prepared at a fixed mixing ratio, using the total nitrogen concentration of the receiving river water at each pollution-source outfall as the reference value; the specific ratios are provided in Table S2.

Subsamples were collected at 0, 1, 3, 12, and 24 h for physicochemical analyses. Microbial, metagenomic, transcriptional, and rate measurements focused on the key response windows at 3, 12, and 24 h.

### 2.2. Physicochemical Parameters and Dissolved Metals

Water samples were filtered through pre-rinsed 0.45 μm membranes prior to chemical analyses. Dissolved oxygen (DO), pH, and temperature were measured using a portable multiparameter meter (HQ40d, HACH, Loveland, CO, USA). Chemical oxygen demand (COD) was determined by the dichromate oxidation method. Total nitrogen (TN) and total phosphorus (TP) were measured after alkaline persulfate digestion followed by colorimetric determination. Ammonium (NH_4_^+^-N) was determined by the indophenol blue method, phosphate (PO_4_^3−^-P) by the molybdenum blue method, and nitrate (NO_3_^−^-N) and sulfate (SO_4_^2−^) by ion chromatography (ICS-6000, Thermo Fisher Scientific, Sunnyvale, CA, USA). Spectrophotometric analyses were performed using a UV-2600 spectrophotometer (Shimadzu, Kyoto, Japan).

To describe the relative balance between organic loading and nitrate availability, a COD-to-NO_3_^−^-N ratio was calculated as a proxy index.

For dissolved metal analyses, filtered water samples were acidified to pH < 2 with ultrapure HNO_3_ immediately after collection. Concentrations of Fe, Mn, Cu, Zn, and Pb were determined using ICP-MS (7900, Agilent Technologies, Santa Clara, CA, USA). Multi-element standards, procedural blanks, and certified reference materials were included for quality control.

### 2.3. DNA Extraction, 16S rRNA Gene Sequencing, and Community Analysis

For bacterial community analysis, water samples were filtered through sterile 0.22 μm polycarbonate membranes, and total DNA was extracted using the DNeasy PowerWater Kit (QIAGEN, Hilden, Germany) according to the manufacturer’s instructions. DNA quality and concentration were checked using a NanoDrop 2000 spectrophotometer (Thermo Fisher Scientific, Wilmington, DE, USA) and agarose gel electrophoresis.

The V3–V4 region of the bacterial 16S rRNA gene was amplified using primers 338F and 806R. PCR products were purified, pooled in equimolar concentrations, and sequenced on an Illumina MiSeq platform (PE300; Illumina, San Diego, CA, USA).

Raw reads were processed in QIIME2 using DADA2 for quality filtering, denoising, paired-end merging, and chimera removal, generating amplicon sequence variants (ASVs). Taxonomy was assigned against the SILVA database (version 138). All samples were rarefied to the same sequencing depth prior to downstream analyses.

Alpha diversity was evaluated using the Chao1 and Shannon indices. Community dissimilarity was calculated using Bray–Curtis distances and visualized by non-metric multidimensional scaling (NMDS). Relative abundances of dominant taxa were summarized at the genus level.

### 2.4. Metagenomic Sequencing and Annotation of Nitrogen-Cycling Genes

Metagenomic analyses were conducted on biomass collected on 0.22 μm filters at 3, 12, and 24 h. Libraries were prepared following the Illumina standard protocol and sequenced on the Illumina NovaSeq 6000 platform (PE150; Illumina, San Diego, CA, USA).

Raw reads were quality-filtered using fastp and assembled *de novo* using MEGAHIT. Open reading frames were predicted with Prodigal, and a non-redundant gene catalog was constructed using CD-HIT at 95% nucleotide identity. Clean reads were mapped back to the gene catalog to estimate gene abundance. Functional annotation was performed against the KEGG and NCycDB databases using DIAMOND with an E-value cutoff of 1 × 10^−5^.

Nitrogen-cycling genes were assigned to major pathways, including nitrification (*amoA (ammonia monooxygenase subunit A)*, *amoB (ammonia monooxygenase subunit B)*, *amoC (ammonia monooxygenase subunit C)*, *hao (hydroxylamine oxidoreductase)*, *nxrA (nitrite oxidoreductase subunit A)*, and *nxrB (nitrite oxidoreductase subunit B)*), denitrification (*napA (periplasmic nitrate reductase catalytic subunit A)*, *napB (periplasmic nitrate reductase subunit B)*, *nirK (copper-containing nitrite reductase)*, *nirS*, *norB (nitric oxide reductase subunit B)*, *norC (nitric oxide reductase subunit C)*, and *nosZ*), DNRA (*napA*, *napB*, *narH (respiratory nitrate reductase subunit beta)*, *narI (respiratory nitrate reductase subunit gamma)*, *nirB (nitrite reductase [NADH] large subunit)*, *nrfA*, and *nirA (ferredoxin-nitrite reductase)*), and anammox (*hzs (hydrazine synthase)* and *hdh (hydrazine dehydrogenase)*). The relative abundance of each target gene was calculated as its proportion of the total annotated nitrogen-cycling gene abundance in each sample.

### 2.5. RNA Extraction and RT-qPCR

To validate transcriptional responses of key nitrogen-cycling genes, parallel samples collected at 3, 12, and 24 h were filtered through sterile 0.22 μm membranes for RNA extraction. Total RNA was extracted using the RNeasy PowerWater Kit (QIAGEN, Hilden, Germany), and residual genomic DNA was removed with the RNase-Free DNase Set (QIAGEN, Hilden, Germany). RNA quality was assessed by NanoDrop 2000 and agarose gel electrophoresis. First-strand cDNA was synthesized using the PrimeScript RT reagent Kit with gDNA Eraser (Takara Bio, Dalian, China).

Transcript abundances of *amoA*, *nirS*, *nosZ*, and *nrfA* were quantified by reverse transcription quantitative PCR (RT-qPCR) using published primers (Table S1) and TB Green Premix Ex Taq II (Takara Bio, Dalian, China) on a CFX96 Real-Time PCR Detection System (Bio-Rad, Hercules, CA, USA). The thermal profile consisted of 95 °C for 3 min, followed by 40 cycles of 95 °C for 10 s, 60 °C for 30 s, and 72 °C for 30 s, followed by melt-curve analysis. All reactions were run in triplicate with no-template controls.

These four genes were selected as representative transcriptional markers because *amoA* represents the entry step of nitrification, *nirS* represents nitrite reduction within denitrification, *nosZ* represents the terminal reduction of nitrous oxide to dinitrogen, and *nrfA* is the key functional marker for DNRA-mediated nitrite reduction to ammonium.

Relative transcript levels were calculated using the 2^−ΔΔCt^ method, with the source water control (YS) used as the calibrator at each time point and 16S rRNA transcripts used for normalization.

### 2.6. Nitrogen Transformation Rate Measurements

Potential DNRA and denitrification rates were determined by short-term ^15^N tracer incubations. At 3, 12, and 24 h, subsamples from each treatment were transferred into gas-tight serum bottles and amended with K^15^NO_3_ (98 atom%) to enrich the nitrate pool without substantially altering background nitrogen conditions. Incubations were conducted in the dark under the same temperature conditions as the microcosm experiment.

Denitrification rates were determined from the production of ^29^N_2_ and ^30^N_2_ after ^15^NO_3_^−^ amendment using IRMS.

DNRA rates were determined from the production of ^15^NH_4_^+^ after ^15^NO_3_^−^ amendment using the microdiffusion method followed by IRMS analysis. Procedural blanks were included in each analytical batch, and blank-corrected values were used for final calculations. The analytical detection threshold was evaluated based on procedural blank variability, and ^15^N tracer recovery was assessed by isotope mass balance across the measured product pools.

Potential N_2_O production rates were measured in parallel sealed incubations with defined headspace volume. Gas samples were analyzed using a gas chromatograph equipped with an electron capture detector (GC-2014, Shimadzu, Kyoto, Japan). Dissolved N_2_O was corrected using the Bunsen solubility coefficient, and rates were expressed as nmol N_2_O L^−1^ h^−1^.

### 2.7. Statistical Analysis

All data are presented as mean ± standard deviation (SD). Differences among treatments within the same time point were tested using one-way analysis of variance (ANOVA) followed by Tukey’s HSD test when assumptions of normality and homogeneity of variance were met; otherwise, the Kruskal–Wallis test followed by Dunn’s post hoc test was applied. Statistical significance was set at *p* < 0.05.

Spearman correlation analysis was used to examine relationships among environmental variables. Mantel tests were performed to evaluate associations between nitrogen-cycling functional matrices and individual environmental distance matrices using 9999 permutations. Redundancy analysis (RDA) was used to assess relationships between nitrogen transformation processes and environmental variables. Prior to RDA, process data were Hellinger-transformed, environmental variables were *z*-score standardized, and collinearity was screened using variance inflation factors (VIF < 10).

Potential ecological functions of bacterial communities were inferred using the FAPROTAX database based on taxonomic assignments. Functional groups related to carbon and nitrogen cycling, including chemoheterotrophy, aerobic chemoheterotrophy, fermentation, nitrate reduction, and host-associated functions, were extracted and compared as relative abundances. Because FAPROTAX provides taxonomy-based functional inference, these results were interpreted only as supplementary evidence together with metagenomic, transcriptional, and process-rate data. All analyses were conducted in R 4.3.1 using the packages *vegan*, *ggplot2*, *linkET*, *reshape2*, and *dplyr*.

## 3. Results

### 3.1. Source-Specific Inputs Established Distinct Hydrochemical Backgrounds

**Figure 1:**
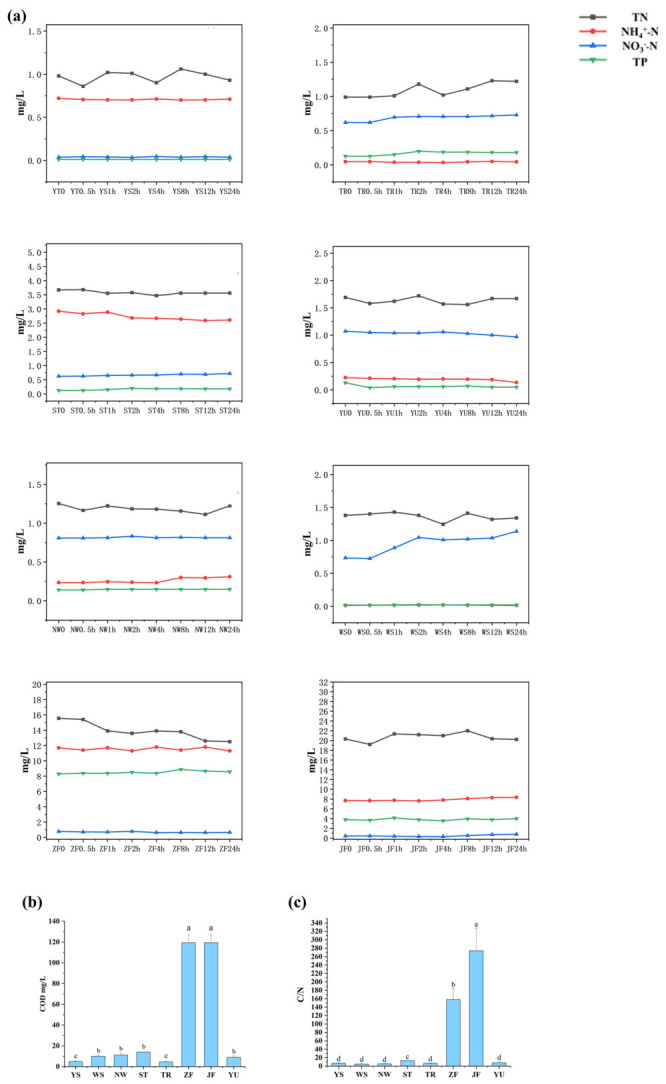
Hydrochemical responses of river water to different nitrogen-source inputs. (**a**) Temporal changes in TN, NH_4_^+^-N, NO_3_^−^-N, and TP during the 0–24 h incubation. (**b**) COD concentrations under different input scenarios. (**c**) COD-to-NO_3_^−^-N ratio under different input scenarios. YS, source water; WS, wastewater treatment plant effluent; ST, agricultural tailwater; YU, aquaculture effluent; NW, rural domestic sewage; TR, soil runoff; ZF, pig manure; and JF, chicken manure. Different lowercase letters indicate significant differences among treatments (*p* < 0.05).

As shown in Figure 1, different nitrogen-source inputs rapidly established distinct hydrochemical conditions within 0–24 h. The manure-related treatments (ZF and JF) consistently showed the highest TN, NH_4_^+^-N, and COD concentrations, together with markedly elevated COD-to-NO_3_^−^-N ratios, indicating a carbon-rich and strongly reducing background. In contrast, wastewater treatment plant effluent (WS) and rural domestic sewage (NW) were characterized by moderate organic loading but relatively high inorganic nitrogen availability, with WS showing particularly high NO_3_^−^-N levels. The agricultural tailwater treatment (ST) exhibited relatively high nitrogen loading and moderately elevated COD, representing an intermediate condition between sewage-related and manure-related inputs. By comparison, YU and TR remained at low to intermediate levels, whereas YS consistently maintained the lowest nutrient and organic matter concentrations. Overall, the different source inputs created a clear gradient from low-load background conditions to high organic and nitrogen loading conditions, thereby providing the hydrochemical basis for subsequent divergence in nitrogen-cycling pathways.

### 3.2. Microbial Communities Were Restructured in a Source-Dependent Manner

**Figure 2:**
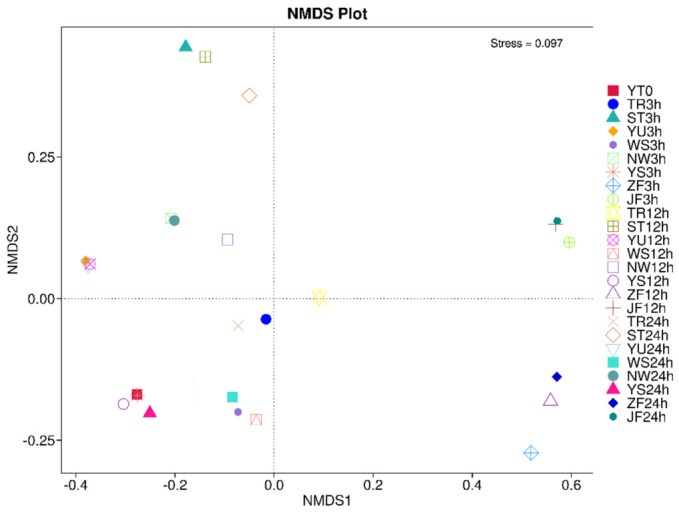
Non-metric multidimensional scaling (NMDS) ordination based on Bray–Curtis dissimilarity of microbial community composition across treatments. Stress = 0.097.

As shown in Figure 2, microbial communities were rapidly restructured under different nitrogen-source inputs. The NMDS ordination showed clear source-dependent separation of samples, with a low stress value (0.097), indicating a reliable representation of community dissimilarity. The YS samples clustered tightly, suggesting relative stability of the background community, whereas all input treatments shifted away from the YS cluster to varying degrees. The manure-related treatments (ZF and JF) showed the largest displacement and were clearly separated from the non-manure groups, while WS, NW, ST, and TR also displayed distinct deviations from the background condition.

Community composition supported the ordination pattern. YS remained dominated by typical freshwater taxa such as *Polynucleobacter* and *Limnohabitans*, whereas WS and NW showed increasing relative abundance of *Escherichia-Shigella*, indicating the introduction of sewage-associated microbial signatures. The strongest restructuring occurred in ZF and JF, where gut-associated anaerobic or facultatively anaerobic genera, including *Lactobacillus*, *Clostridium sensu stricto 1*, *Bacteroides*, *Fusobacterium*, and *Prevotella*, became enriched. Together, these results indicate that different source inputs rapidly generated distinct microbial assemblages and that manure-related inputs caused the greatest departure from the native freshwater community.

### 3.3. Metagenomic Profiles Revealed Source-Dependent Redistribution of Nitrogen-Cycling Pathways

**Figure 3:**
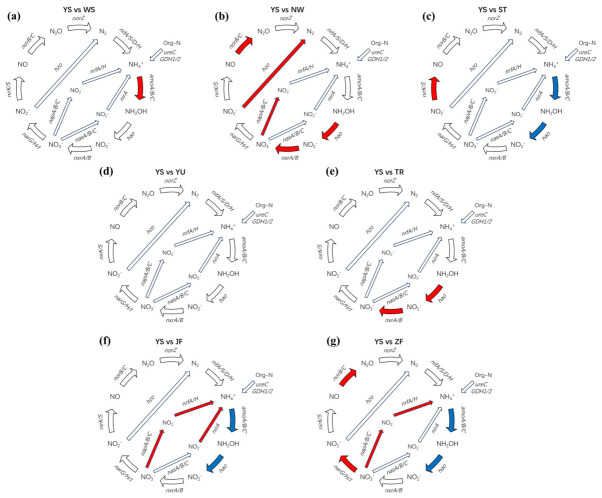
Source-dependent redistribution of nitrogen-cycling functional modules relative to the source-water control (YS). Panels (**a**–**g**) compare YS with WS, NW, ST, YU, TR, JF, and ZF, respectively. Red indicates a significant increase relative to YS, blue indicates a significant decrease, and white indicates no significant change. Statistical comparisons were conducted between each treatment and YS using one-way ANOVA (*p* < 0.05). *N/A* indicates that the gene abundance was below the detection threshold.

As shown in Figure 3, nitrogen-cycling functional modules displayed clear source-dependent redistribution patterns when compared with the source water background. Different inputs affected the nitrification chain, the entry of nitrate reduction, the intermediate steps of denitrification, and the ammonium-producing branch in distinct ways, indicating that external nitrogen sources not only altered substrate conditions, but also reshaped the overall structure of nitrogen transformation pathways.

The wastewater treatment plant effluent treatment (WS) was mainly characterized by enhancement of the nitrification entry step. The *amo* module showed a consistent upward response, whereas nitrate reduction and denitrification modules did not exhibit a comparable overall increase. This pattern suggests that WS primarily strengthened the oxidative branch of nitrogen cycling, likely by favoring ammonia oxidation rather than broadly stimulating reductive pathways.

The rural domestic sewage treatment (NW) showed coordinated changes across multiple pathway modules. The nitrate reduction entry module (*nap*) increased, indicating enhanced nitrate supply to downstream reductive pathways. At the same time, the denitrification intermediate module (*nor*) and the nitrification-related *hao* and *nxr* modules were also elevated. This combined pattern suggests that sewage-related input promoted simultaneous adjustment of both oxidative and reductive processes, resulting in a more tightly coupled nitrogen transformation network.

The agricultural tailwater treatment (ST) showed a different response pattern, with reduced nitrification-related modules (*amo* and *hao*) but enhanced reductive entry into the denitrification chain. This indicates that agricultural input tended to weaken the oxidative nitrogen transformation route while increasing the relative contribution of nitrate reduction, thereby shifting nitrogen turnover toward denitrification-related pathways. In contrast, the soil runoff treatment (TR) mainly enhanced the downstream nitrification modules, particularly *hao* and *nxr*, suggesting greater continuity of the nitrification chain and a tendency toward nitrate production rather than nitrate reduction.

By comparison, the aquaculture-related treatment (YU) showed largely neutral responses, with most modules remaining unchanged. This indicates that, within the experimental window, aquaculture input had a comparatively limited net effect on pathway-level nitrogen cycling or that opposing responses among individual steps offset each other at the module scale.

The manure-related treatments showed the clearest shift toward ammonium-producing pathways. In both JF and ZF, the nitrate reduction entry module (*nap*) and the DNRA-related modules (*nrf* and *nirA*) were enhanced, whereas the nitrification entry modules (*amo* and *hao*) were suppressed. These responses indicate that manure inputs redirected nitrate reduction toward ammonium regeneration while reducing the contribution of oxidative nitrification. A difference was also observed between the two manure types: ZF showed a more apparent increase in the *nor* module, whereas the corresponding response in JF was weaker. Overall, these results demonstrate that manure-related inputs favored nitrogen retention through DNRA-type pathways, whereas wastewater- and sewage-related inputs were more likely to maintain or enhance oxidative and denitrification-linked transformations.

### 3.4. Key Nitrogen-Cycling Functional Genes Showed Source-Dependent Differentiation

Detailed gene-level profiles showed that the relative abundances of key nitrogen-cycling functional genes varied markedly among different nitrogen-source inputs, indicating strong source-dependent shifts in the functional structure of the nitrogen cycle. Overall, genes involved in nitrification remained relatively abundant under wastewater treatment plant effluent input, whereas manure-related inputs strongly suppressed nitrification-associated functions. Denitrification-related genes were generally more abundant under sewage-related inputs, although the responses of individual steps within the denitrification pathway were not always synchronized. In contrast, key DNRA genes were mainly enriched in the manure-input groups, while anammox-related genes showed a relatively clear enrichment signal only in the rural domestic sewage treatment. These results suggest that different source inputs did not simply enhance or inhibit a single nitrogen transformation process; rather, they systematically altered the relative contributions of different branches within the nitrogen-cycling network, particularly the fate of nitrate reduction and the coupling between oxidative and reductive pathways.

Changes in nitrification-related genes most clearly reflected the divergence in oxidative nitrogen transformation capacity among treatments. The YS group maintained stable and detectable relative abundances of nitrification-related genes throughout the 0–24 h incubation, indicating that the background water retained an intrinsic nitrification potential. The WS group consistently remained at a relatively high level, particularly during the early incubation stage, suggesting that wastewater effluent input enhanced the contribution of nitrification-related functional groups. The NW group showed an intermediate level but declined over time, implying a transient nitrification potential during the short-term response. In contrast, the relative abundances of nitrification-related genes in the ZF and JF groups were nearly zero, indicating that manure inputs strongly inhibited the nitrification chain. A more detailed examination of individual genes further showed that *amoA*, *amoB*, *amoC*, *nxrA*, and *nxrB* remained relatively abundant at multiple time points in WS, whereas ammonia-oxidation-related genes in the manure groups were consistently maintained at very low levels. Only some downstream genes, such as *nxrA* and *nxrB*, remained detectable in the middle to late incubation period, suggesting that the upstream and downstream steps of the nitrification chain were not necessarily synchronized under different source-input scenarios, and that ammonia oxidation was more likely to constitute the limiting step.

Compared with nitrification, denitrification-related genes showed stronger modular differentiation. The overall relative abundance of denitrification-related genes was higher in WS and NW than in most other treatments, with WS remaining at a relatively high level at 3, 12, and 24 h, indicating that sewage-related inputs were more favorable for maintaining a relatively large denitrification gene pool. A more detailed breakdown of the denitrification pathway further revealed that different steps responded in distinct ways. The nitrate-reduction entry genes *napA* and *napB* were in fact highest in ZF and JF and further increased from 12 to 24 h, indicating that manure inputs enhanced the entry potential of nitrate reduction to nitrite. In contrast, the downstream nitrite-reduction gene *nirK* and the terminal nitrous oxide reduction gene *nosZ* remained relatively abundant in TR, ST, and NW, but were overall lower in ZF and JF. This indicates that although manure inputs strengthened the entry step of nitrate reduction, they did not synchronously enhance the downstream steps required for complete denitrification. Meanwhile, *norB* remained relatively abundant in NW and WS, suggesting that the intermediate conversion from NO to N_2_O was more readily activated under sewage-related inputs. Taken together, these patterns suggest that sewage-derived inputs were more likely to maintain a relatively complete denitrification chain, whereas manure-related inputs showed a pattern of enhanced pathway entry but incomplete downstream coupling.

Changes in DNRA-related genes further revealed clear divergence in the fate of nitrate reduction. The key DNRA gene *nrfA* exhibited markedly higher relative abundance only in ZF and JF and remained at high levels from 12 to 24 h, whereas it stayed at low levels in all other treatments. This strongly suggests that manure inputs enhanced the functional potential for nitrite reduction to ammonium, thereby redirecting nitrate reduction toward the DNRA pathway rather than toward gaseous nitrogen production. It is noteworthy that entry-associated genes such as *napA*, *napB*, *narH*, and *narI* remained detectable in multiple treatments, indicating that the potential for nitrate-to-nitrite reduction was widespread across source-input scenarios. Thus, the key determinant of nitrogen fate was not whether nitrate reduction occurred, but rather how the downstream balance shifted between *nrfA*-mediated ammonium regeneration and denitrification-related genes. The combination of high *nrfA*, low *nosZ*, and suppressed nitrification genes in the manure groups therefore suggests that nitrate reduction under these conditions was preferentially redistributed toward nitrogen retention.

Anammox-related genes showed the narrowest response range but still exhibited some degree of source specificity. The *hzs* and *hdh* genes were nearly absent or remained at extremely low levels in all treatments except NW during 3–24 h, indicating that anammox was not a major potential pathway under most source-input scenarios. By contrast, NW showed a relatively high *hzs* signal as early as 3 h, while *hdh* further increased at 12–24 h, suggesting that rural domestic sewage input may have provided a more suitable substrate background and microenvironment for anammox, thereby allowing this process to make a measurable contribution during short-term incubation. Nevertheless, given the overall abundance pattern, anammox should still be regarded as a secondary pathway in this system, with its ecological significance mainly reflected in localized enhancement under specific sewage-related input conditions rather than as a dominant nitrogen removal pathway.

Overall, the functional gene patterns indicate that the most important difference among source-input scenarios did not lie in whether nitrate-reduction potential existed, but rather in how nitrate reduction was partitioned downstream. Wastewater treatment plant effluent and rural domestic sewage generally maintained relatively high nitrification- and denitrification-related functional potential, suggesting stronger coupling between oxidative processes and nitrogen-removal pathways. In contrast, manure inputs strongly suppressed nitrification entry while increasing the contribution of DNRA-related genes such as *nrfA*, thereby redirecting nitrate reduction toward ammonium regeneration and promoting nitrogen retention. Agricultural tailwater and soil runoff mainly showed localized enhancement of specific pathway components, whereas aquaculture-related input exerted a comparatively limited effect on the overall pathway structure. These functional-gene results provide direct support for the subsequent RT-qPCR and process-rate measurements.

### 3.5. Transcriptional Responses of Key Nitrogen-Cycling Genes Further Supported Source-Dependent Pathway Allocation

**Figure 4:**
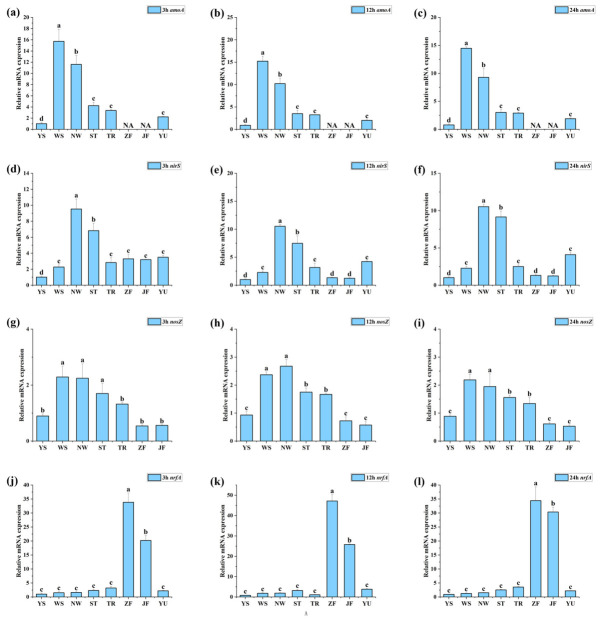
Transcriptional responses of key nitrogen-cycling genes quantified by RT-qPCR under different nitrogen-source inputs. Panels (**a**–**c**) show *amoA* at 3, 12, and 24 h; panels (**d**–**f**) show *nirS* at 3, 12, and 24 h; panels (**g**–**i**) show *nosZ* at 3, 12, and 24 h; and panels (**j**–**l**) show *nrfA* at 3, 12, and 24 h. Bars represent means ± SD. Different letters indicate significant differences among treatments within the same time point (*p* < 0.05).

As shown in Figure 4, different nitrogen-source inputs triggered rapid transcriptional responses of key nitrogen-cycling genes at the early stage of incubation, and these responses remained clearly source-dependent at 12 and 24 h. This pattern indicates that external inputs not only altered the composition of nitrogen-cycling functional genes, but also directly drove pathway differentiation at the transcriptional level. Overall, sewage-related inputs were more likely to sustain high expression of genes involved in nitrification and denitrification, whereas manure-related inputs more strongly enhanced the transcriptional activity of the key DNRA gene, indicating that distinct source types rapidly generated clearly differentiated functional-response patterns.

Specifically, *amoA* remained at relatively high levels in WS and NW throughout the incubation and already showed a clear advantage at 3 h, indicating that sewage-related inputs rapidly activated ammonia oxidation, the initial step of nitrification, and maintained relatively high transcriptional activity thereafter. By comparison, the YS group remained at an overall low level, while ST and TR showed only intermediate expression. In contrast, *amoA* was not detected in either ZF or JF at any of the three time points, suggesting that manure inputs strongly suppressed the transcriptional response of the nitrification entry step. This pattern was consistent with the metagenomic results, in which nitrification-related functional genes were markedly reduced in the manure treatments.

The expression of denitrification-related genes showed a more distinct source-dependent pattern. *nirS* remained highly expressed in NW and ST throughout the incubation, with relatively stable variation over time, indicating that rural domestic sewage and agricultural tailwater inputs more readily stimulated the early denitrification step. Meanwhile, *nosZ* remained relatively high in WS and NW and reached its highest level at 12 h, suggesting that sewage-related inputs not only enhanced the upstream denitrification response, but also maintained a relatively strong transcriptional potential for the terminal reduction of N_2_O. In contrast, *nosZ* remained consistently low in ZF and JF, indicating that the terminal step of denitrification contributed less under manure-input conditions.

Compared with the above genes, the DNRA marker gene *nrfA* showed the strongest source specificity. Both ZF and JF exhibited expression levels far higher than those of the other treatments as early as 3 h, reached peak values at 12 h, and remained high at 24 h. This result indicates that manure-related inputs rapidly and persistently activated the transcriptional process of nitrite reduction to ammonium. In all other treatments, *nrfA* remained at generally low levels, suggesting that transcriptional enhancement of DNRA was largely restricted to the manure-input scenarios characterized by high organic loading. Together with the long-term absence of detectable *amoA* signals in the manure groups, these results indicate that nitrogen cycling under manure input was shifted away from oxidative nitrification and toward reductive ammonium regeneration.

### 3.6. Nitrogen Transformation Rates Validated Source-Dependent Pathway Allocation

**Figure 5:**
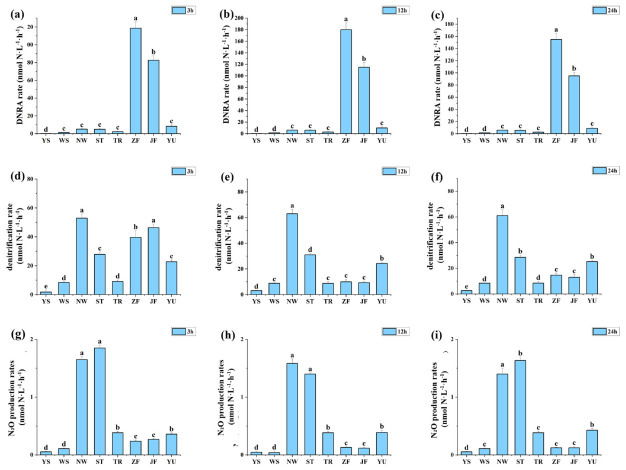
Nitrogen transformation rates under different nitrogen-source inputs. Panels (**a**–**c**) show DNRA rates at 3, 12, and 24 h; panels (**d**–**f**) show denitrification rates at 3, 12, and 24 h; and panels (**g**–**i**) show N_2_O production rates at 3, 12, and 24 h. Bars represent means ± SD. Different letters indicate significant differences among treatments within the same time window (*p* < 0.05).

As shown in Figure 5, nitrogen transformation rates displayed clear source-dependent differences across the 3–24 h incubation. DNRA rates were highest in ZF and JF, particularly at 12 h and 24 h, confirming that manure-related inputs redirected nitrate reduction toward ammonium regeneration. In contrast, denitrification rates were generally higher in WS, NW, and ST, indicating that sewage-related and agricultural inputs more strongly supported removal-oriented nitrate consumption. N_2_O production was also elevated in WS and NW and remained high in some treatments at 12 h, suggesting that sewage-related inputs enhanced denitrification but simultaneously increased byproduct risk.

These rate measurements were consistent with the metagenomic and transcriptional results. Treatments characterized by high *nrfA* abundance and expression also showed higher DNRA rates, whereas treatments with stronger *nirS* and *nosZ* responses were more closely associated with denitrification. Overall, the process-based evidence confirms that 12 h represented a critical window for source-dependent pathway redistribution and that different nitrogen sources shifted nitrate reduction toward either nitrogen retention or nitrogen removal.

### 3.7. Environmental Gradients Governed Nitrogen Pathway Partitioning

**Figure 6:**
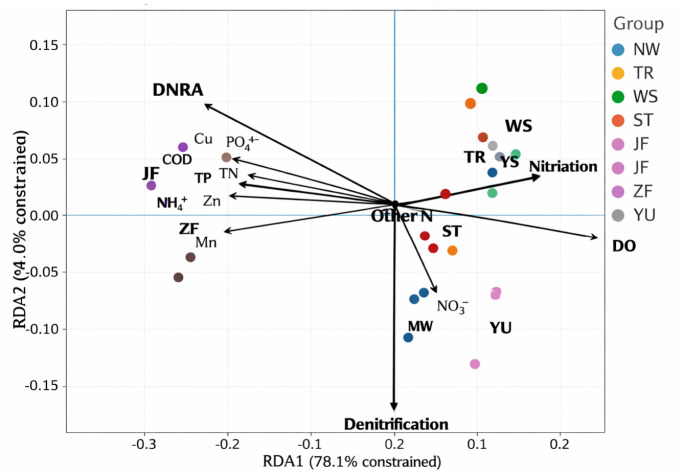
Redundancy analysis (RDA) of nitrogen transformation processes and environmental variables.

As shown in Figure 6, the RDA revealed clear associations between nitrogen transformation processes and environmental gradients under different source-input scenarios. RDA1 and RDA2 explained 78.1% and 14.0% of the constrained variation, respectively, indicating that the major differences among treatments were primarily distributed along the first ordination axis, while the second axis further separated variation related to different nitrate-reduction outcomes. Overall, the ordination pattern showed that different source inputs generated distinct environmental backgrounds, which were closely linked to shifts in nitrogen pathway structure. All retained environmental variables showed acceptable collinearity levels (VIF < 10; Table S3), supporting the robustness of the final ordination model.

JF and ZF were mainly distributed on the negative side of RDA1 and were closely associated with the vectors of COD, NH_4_^+^-N, TN, TP, PO_4_^3−^-P, and the dissolved metals Cu, Zn, and Mn. This indicates that the manure-input treatments were characterized by high organic loading, elevated nutrient concentrations, and co-occurring metal enrichment. In contrast, YS, WS, and TR were more frequently positioned on the positive side of RDA1 and aligned more closely with the DO vector, suggesting that these treatments were generally associated with relatively more oxidizing conditions. NW and YU showed a more pronounced shift along the negative direction of RDA2 and were located closer to the NO_3_^−^-N vector, implying stronger association with nitrate-rich conditions. ST was located near the central part of the ordination space, indicating an intermediate environmental background between the more oxidizing and more reducing treatment groups.

The process vectors further illustrated the divergence of nitrogen-cycling pathways along these gradients. The nitrification vector was oriented toward the positive side of RDA1 and closely followed the direction of DO, indicating that nitrification contributed more strongly under relatively oxic conditions. Its opposite position relative to COD and NH_4_^+^-N suggests that oxidative nitrogen transformation weakened as organic loading and reduced nitrogen increased. In contrast, the DNRA vector was located on the negative side of RDA1 and showed closer alignment with TN, NH_4_^+^-N, TP, PO_4_^3−^-P, and some metal vectors, indicating that DNRA was favored under more nutrient-enriched, organic-rich, and reducing conditions. The close proximity of JF and ZF to the DNRA vector, together with their separation from the nitrification vector, further supports a marked shift toward reductive nitrogen retention under manure input.

The denitrification vector was mainly distributed along the negative direction of RDA2 and was more closely aligned with the NO_3_^−^-N vector, suggesting that denitrification was more strongly associated with nitrate availability. NW and YU were positioned closer to this vector, indicating that under these input scenarios nitrate reduction was more likely to proceed toward denitrification-related removal processes, or at least toward a process structure with a higher contribution of denitrification. Other process vectors were located closer to the center of the ordination space, suggesting relatively weaker treatment separation or stronger joint regulation by multiple environmental factors.

## 4. Discussion

### 4.1. Organic Loading and Nitrate Availability Jointly Regulate Competition Between DNRA and Denitrification

One of the central findings of this study is that the fate of nitrate reduction was not determined simply by the presence or absence of nitrate-reducing potential, but by how organic loading and nitrate availability jointly structured competition between DNRA and denitrification. Across the different source-input scenarios, manure-related treatments consistently exhibited the highest COD, NH_4_^+^-N, and COD-to-NO_3_^−^-N ratios, and these hydrochemical features were accompanied by elevated *nrfA* abundance and transcription, together with markedly enhanced DNRA rates. In contrast, sewage-related and agricultural tailwater inputs maintained comparatively higher nitrate availability and a more moderate organic-loading background, under which denitrification-related genes and denitrification rates were more strongly expressed. This contrast indicates that external nitrogen sources affected not only the magnitude of nitrate reduction, but also its downstream partitioning between nitrogen retention and nitrogen removal [1,2,6]. It should also be noted that DNA-based gene abundance, transcript abundance, and measured process rates are not expected to exhibit strict one-to-one correspondence, because they represent different levels of functional organization, namely pathway potential, short-term transcriptional activation, and net process performance.

From a mechanistic perspective, the dominance of DNRA under manure input is consistent with resource-ratio theory and with the electron donor–electron acceptor framework widely used to explain nitrate reduction partitioning. Under carbon-rich and relatively nitrate-limited conditions, DNRA can become thermodynamically and ecophysiologically advantageous because it conserves more reducing power per mole of nitrate reduced and is therefore favored when electron donors are abundant relative to nitrate [2,7,9]. In the present study, the COD-to-NO_3_^−^-N ratio in the manure treatments was substantially higher than that in the other groups, and this high-carbon background coincided with the near disappearance of *amoA* signals, strong upregulation of *nrfA*, and the highest DNRA rates at 12 h and 24 h. These results suggest that manure input did not merely suppress oxidative nitrogen transformation, but actively redirected nitrate reduction toward ammonium regeneration. By contrast, in WS, NW, and partly ST, nitrate remained relatively more available and the organic-loading background was less extreme, which appears to have supported denitrification-linked nitrate consumption rather than DNRA-dominated nitrogen retention [2,6,7]. The markedly higher COD:NO_3_^−^-N ratios in the manure treatments further support this resource-ratio interpretation, indicating a carbon-rich and relatively nitrate-limited background more favorable to DNRA than to denitrification.

Another important implication of our results is that nitrate availability did not simply promote all nitrate-reduction pathways equally, but appeared to bias the system toward denitrification when combined with a less reducing hydrochemical background. In the sewage-related treatments, particularly NW, nitrate availability remained relatively high, while *nirS* and *nosZ* expression and denitrification rates were consistently elevated. This pattern suggests that when nitrate supply is sufficient and organic loading is not excessively skewed toward strongly reducing conditions, nitrate is more likely to flow through denitrification-linked removal pathways. This interpretation is supported by previous studies showing that high nitrate availability often favors denitrification over DNRA, whereas DNRA tends to gain a relative advantage when nitrate becomes limiting but electron donors remain abundant [2,3,6,10]. In our system, the RDA further supported this interpretation by showing that denitrification was more closely aligned with NO_3_^−^-N, whereas DNRA was more closely associated with COD, NH_4_^+^-N, TP, PO_4_^3−^-P, and some dissolved metals. Thus, the contrast between manure- and sewage-related inputs should not be interpreted as a simple difference in “pollution severity,” but rather as a difference in how substrate stoichiometry and redox background reshape nitrate fate [3,6,10].

The temporal dimension of our results is also noteworthy. The divergence between DNRA- and denitrification-dominated structures emerged rapidly, and 12 h appeared to represent a critical transition window. In the manure treatments, *nrfA* transcription peaked at 12 h and DNRA rates simultaneously reached their highest values, suggesting that once a strongly reducing and carbon-rich microenvironment had been established, nitrate reduction was quickly redirected toward ammonium regeneration. In contrast, denitrification and N_2_O production in NW and ST were already high at 3 h and remained elevated thereafter, indicating that denitrification-linked responses can be activated very early when nitrate supply is sufficient and redox conditions remain compatible with partial or relatively complete denitrification. This rapid differentiation highlights that the effects of pollution-source inputs on nitrate fate may occur on timescales much shorter than those usually captured by routine monitoring, and that short-term pulses of carbon-rich waste may have disproportionate effects on nitrogen retention [10,11].

A more mechanistic interpretation of the 12 h threshold is that pathway redistribution likely emerged from the combined effects of rapid oxygen depletion, substrate reallocation, and short-term microbial functional adjustment after source input. Under high organic loading, heterotrophic respiration can quickly intensify oxygen consumption and generate low-oxygen microniches, even when the bulk water is not completely anoxic, thereby suppressing nitrification while favoring nitrate-reduction pathways. At the same time, the competition between DNRA and denitrification is expected to shift dynamically as nitrate and organic carbon are consumed and redistributed during incubation, rather than remaining constant through time. In addition, transcriptional responses of nitrogen-cycling genes can occur within only a few hours after abrupt environmental change, indicating that functional reorganization may precede longer-term shifts in community structure. In our system, the pronounced divergence at 12 h therefore likely reflects the point at which early geochemical restructuring and rapid gene-level responses became sufficiently amplified to produce a clear shift in nitrate-reduction fate [5,10,11].

These findings have important implications for how nitrogen pollution is interpreted and managed in river systems. Conventional water-quality assessment often emphasizes TN concentration, yet our results suggest that similar nitrogen loads may lead to very different ecological outcomes depending on whether nitrate reduction ends in gaseous loss or ammonium regeneration. From this perspective, manure-related inputs may be particularly problematic because they not only increase nitrogen loading, but also create conditions that favor DNRA and therefore retain reactive nitrogen within the aquatic system. Such nitrogen retention can prolong eutrophication pressure and sustain subsequent internal cycling, even when external nitrate is being reduced [1,10]. By contrast, sewage-related inputs may support stronger denitrification, but this does not necessarily imply a benign outcome, because enhanced denitrification can also coincide with elevated N_2_O production when upstream and terminal steps are not fully balanced [12]. Therefore, source-oriented nitrogen management should move beyond total nitrogen control and pay closer attention to the hydrochemical conditions that determine nitrate fate, particularly organic loading, nitrate availability, and redox status [1,2,12].

Taken together, our results support a process-based interpretation in which organic loading and nitrate availability act as the primary joint controls over nitrate-reduction partitioning, while redox conditions and co-occurring nutrient and metal backgrounds further modulate pathway dominance. Under manure input, high organic loading, elevated ammonium, and a strongly reducing background favored DNRA and nitrogen retention; under sewage-related inputs, relatively higher nitrate availability and less extreme reducing conditions favored denitrification-linked nitrate removal. This source-dependent partitioning provides a mechanistic explanation for why different nitrogen inputs can produce markedly different nitrogen-cycling outcomes, even over a short incubation period [2,6,7,10]. From a Water Research perspective, the key implication is that improving river nitrogen removal efficiency requires not only reducing nitrogen inputs per se, but also preventing hydrochemical conditions that redirect nitrate reduction toward nitrogen-retention pathways [1,2,12].

### 4.2. Manure-Related Inputs Favor Nitrogen Retention Rather than Effective Nitrogen Removal

Manure-related inputs favored N retention rather than net N removal by redirecting nitrate reduction away from complete denitrification and toward DNRA-mediated NH_4_^+^ regeneration. This was supported by the consistently higher COD, NH_4_^+^-N, and COD/NO_3_^−^-N ratios in the manure treatments, the prolonged loss of *amoA*, the strong enrichment and transcriptional activation of *nrfA*, and the highest DNRA rates observed during 12–24 h, whereas neither nitrification initiation nor terminal denitrification was concomitantly enhanced. These patterns suggest that nitrate reduction was preferentially redirected toward NH_4_^+^ regeneration rather than N_2_ production, shifting the system from net N removal to internal N retention, consistent with the functional distinction between DNRA and denitrification pathways [1,6].

This shift was not driven by elevated N concentration alone, but by the broader geochemical conditions created by manure inputs. Relative to sewage-derived inputs, manure typically introduced more labile organic matter, higher NH_4_^+^ loads, and stronger oxygen demand, thereby favoring localized reducing conditions. Under such conditions, nitrification was rapidly suppressed, limiting new NO_3_^−^ production [13], while the existing nitrate/nitrite pool was more readily partitioned to DNRA. Previous studies have demonstrated that DNRA can outcompete denitrification under high organic carbon availability and low-oxygen or reducing conditions [2,7], even when the bulk water remains oxygenated due to the formation of anoxic microzones within organic aggregates or particles [3]. The rapid disappearance of *amoA* together with the pronounced increase in *nrfA* in manure treatments strongly supports this mechanism.

Importantly, manure input did not simply inhibit nitrate reduction, but reconfigured its downstream fate. Although *napA* and *napB* remained detectable, indicating that NO_3_^−^ reduction to NO_2_^−^ was maintained, *nosZ* remained low whereas *nrfA* became dominant. This pattern suggests that nitrate reduction was not terminated, but functionally rerouted away from complete denitrification and toward NH_4_^+^ retention, reflecting shifts in nitrate respiration end products under changing environmental controls [2]. As a result, the reduction network remained active, yet its outcome shifted from permanent N loss to internal recycling, consistent with observations in other N-cycling systems [5,12].

The ecological implication of this shift is substantial. NH_4_^+^ regenerated via DNRA can be repeatedly reused as substrate for nitrification, algal uptake, and internal N cycling, thereby prolonging N residence time and sustaining eutrophication pressure in downstream receiving waters [11]. Accordingly, assessing manure impacts solely on the basis of short-term TN changes may underestimate its impairment of river N removal function, because a considerable fraction of “transformed” N is not eliminated from the system but conserved in a bioavailable form.

Overall, these results demonstrate that manure-related inputs favor N retention over effective N removal by creating carbon-rich, oxygen-depleting conditions that suppress nitrification–denitrification coupling and stimulate DNRA-mediated NH_4_^+^ regeneration. From a management perspective, the key risk of manure pollution lies not only in external N loading itself, but also in its capacity to restructure in situ N cycling toward retention rather than removal.

### 4.3. Sewage-Related Inputs Enhance Denitrification but May Increase N_2_O Emission Risk

Unlike manure-related inputs, which more clearly redirected nitrate reduction toward DNRA, sewage-related inputs produced a more complex outcome. In this study, wastewater treatment plant effluent and rural domestic sewage did not strongly promote ammonium regeneration, but instead sustained denitrification-related pathways. At the same time, however, N_2_O production also increased. Thus, sewage-related inputs did not simply enhance effective nitrogen removal, but rather created a state in which denitrification was stimulated while greenhouse-gas risk increased simultaneously [8,14,15].

This pattern was supported by multiple lines of evidence. WS and NW maintained relatively high abundances of denitrification-related genes, showed elevated expression of *nirS* and *nosZ*, and exhibited comparatively high denitrification and N_2_O production rates. These results indicate that sewage-related inputs favored denitrification, but did not necessarily ensure complete reduction to N_2_. Instead, they appeared to maintain a greater potential for N_2_O accumulation at intermediate steps. Similar patterns have been reported in wastewater-impacted rivers, where elevated N_2_O concentrations and more active N_2_O production are often associated with high nitrate loading, low dissolved oxygen, and intensified benthic or biofilm-associated reactions [8,14,15].

Mechanistically, sewage-related inputs likely created conditions that were sufficient to activate denitrification, but not always sufficient to keep the pathway fully balanced. Unlike manure inputs, these sources did not impose extremely high COD or strongly reducing conditions, and therefore did not strongly favor DNRA. Rather, they supplied relatively high NO_3_^−^-N together with moderate organic carbon, which supported the upstream steps of denitrification. Under these conditions, nitrate reduction can proceed rapidly, while the final reduction in N_2_O to N_2_ may not remain fully synchronized with upstream reactions [14,15,16]. This helps explain why relatively high *nirS*, relatively high *nosZ*, and elevated N_2_O production co-occurred in WS and NW.

The ecological implication is that sewage-related inputs create a clear trade-off. Compared with manure inputs, they are more conducive to denitrification-linked nitrogen removal, but this benefit may be partly offset by greater N_2_O release. Even if N_2_O accounts for only a small fraction of total denitrification products, that fraction can still represent a substantial greenhouse-gas source in high-load river networks [14]. Therefore, the environmental effect of sewage-related inputs should not be judged solely by whether denitrification is enhanced, but also by whether N_2_O emission risk is amplified [8,14,15].

Another notable feature was the rapidity of this response. In both WS and NW, key denitrification genes were already upregulated at 3 h, and denitrification and N_2_O-related processes became active early in the incubation. This suggests that sewage-derived inputs can trigger pathway redistribution within only a few hours, meaning that conventional monitoring at daily or longer intervals may miss short-lived windows of intensified N_2_O production [8,15].

Overall, sewage-related inputs should not be regarded as uniformly beneficial simply because they promote denitrification. Rather, they place the system in a removal–emission trade-off, in which nitrate removal is enhanced but N_2_O release risk also increases. This trade-off is likely to be an important feature of nitrogen cycling in human-impacted river systems [8,14,15,16].

We acknowledge that the static, dark, and unshaken microcosm design does not fully capture hydrodynamic mixing, re-aeration, dilution, and spatial heterogeneity in natural rivers. Thus, our results should be interpreted as comparative pathway responses to contrasting source inputs under controlled short-term conditions, rather than direct simulations of in situ river reaches. Future studies incorporating field observations and more hydrodynamically realistic experimental designs will be necessary to assess the broader applicability of the observed pathway redistribution patterns. The fixed mixing ratios used here should therefore be interpreted as comparative exposure scenarios rather than exact in situ dilution conditions, and source effects would likely be stronger under lower dilution but weaker under greater dilution.

### 4.4. Pathway-Specific Implications for Source-Oriented Nitrogen Management

From a management perspective, the pathway-specific responses observed here indicate that source-oriented control should target not only total nitrogen loading, but also the hydrochemical conditions that favor undesirable nitrogen fates. For manure-derived runoff, mitigation should prioritize reducing the delivery of labile organic matter and NH_4_^+^ before these inputs reach receiving waters, for example through source separation, solid–liquid separation, manure pretreatment, and interception systems such as vegetated buffer zones or treatment wetlands. Such measures would help lower the COD:NO_3_^−^-N ratio and reduce the likelihood of DNRA dominance and nitrogen retention. In contrast, for sewage-related inputs, management should focus on minimizing the co-delivery of nitrate and residual biodegradable organic carbon, while preventing localized oxygen depletion in receiving waters through improved tertiary treatment, reduction in residual organic load, and enhancement of in-stream reaeration or mixing where feasible. Given that pathway redistribution occurred within 12 h, short-term monitoring immediately after discharge events may also be important for detecting transient windows of enhanced N_2_O risk and for evaluating the effectiveness of source-specific interventions.

## 5. Conclusions

This study demonstrates that the ecological consequence of external nitrogen input depends not only on nitrogen loading itself, but also on how source-specific hydrochemical conditions redistribute nitrogen-cycling pathways in receiving waters. Manure-related inputs created carbon-rich, highly reduced conditions characterized by elevated COD, NH_4_^+^-N, and COD-to-NO_3_^−^-N ratios. Under these conditions, nitrification was strongly suppressed, *nrfA* abundance and transcription increased markedly, and DNRA became the dominant nitrate-reduction pathway, indicating that manure inputs favored reactive nitrogen retention rather than effective nitrogen removal. In contrast, sewage-related inputs maintained relatively higher nitrate availability and supported denitrification-linked pathways, as reflected by higher *nirS* and *nosZ* expression and elevated denitrification rates. However, this shift toward removal-oriented pathways was accompanied by increased N_2_O production, indicating a clear removal–emission trade-off. Across treatments, metagenomic profiles, transcriptional responses, and process-rate measurements consistently showed that 12 h represented a critical window for pathway redistribution. Environmental gradient analysis further revealed that DO was the primary factor associated with nitrification, whereas COD, NH_4_^+^-N, phosphorus, and co-occurring metals favored DNRA-related processes, and NO_3_^−^-N was more closely associated with denitrification. Overall, these results show that different nitrogen sources regulate whether nitrate reduction proceeds toward gaseous loss or ammonium regeneration. From a management perspective, improving river nitrogen removal requires not only reducing external nitrogen inputs, but also avoiding hydrochemical conditions that redirect nitrate reduction toward nitrogen-retention pathways or amplify N_2_O emission risk.

## Data Availability

The original contributions presented in this study are included in the article. Further inquiries can be directed to the corresponding authors.

## Supplementary Materials

The following supporting information can be downloaded at https://www.mdpi.com/article/10.3390/biology15100741/s1. Table S1. Physicochemical characteristics of the source water; Table S2. Fixed volumetric mixing ratios of source water (YS) and different pollution-source inputs used in the microcosm experiment; Table S3. Variance inflation factors (VIFs) of environmental variables considered in the redundancy analysis (RDA). Variables with VIF > 10 were excluded from the final model.
