# Supplementary material for: Source-Specific Nitrogen Inputs Are Associated with Pathway Partitioning Between Denitrification and DNRA in River Water
Source: Biology (Basel). 2026 May 8;15(10):741. doi: 10.3390/biology15100741 (PMC13203200; doi:10.3390/biology15100741)
Supplement: Supplementary file 1 [file biology-15-00741-s001.zip › biology-4244112-supplementary.pdf]

Supplementary Material

Table S1. Physicochemical characteristics of the source water

Parameter	Value
Water type	Source water
TN (mg L^{-1})	0.86
NH_4^+-N (mg L^{-1})	0.043
NO_3^--N (mg L^{-1})	0.706
TP (mg L^{-1})	<0.01
PO_4^{3-}-P (mg L^{-1})	<0.01
SO_4^{2-} (mg L^{-1})	3.11
COD (mg L^{-1})	4.00
Fe (mg L^{-1})	0.0226
Cu (mg L^{-1})	<0.00008
Zn (mg L^{-1})	<0.00067
Mn (mg L^{-1})	0.013221
Cd (mg L^{-1})	<0.00005
Pb (mg L^{-1})	0.000514
pH	7.62
T (° C)	15.3
DO (mg L^{-1})	9.13

.

Table S2. Fixed volumetric mixing ratios of source water (YS) and different pollution-source inputs used in the microcosm experiment.

Group	Volume of pollution-source input (L)
TR	50 L
ST	19.8 L
YU	27.7 L
WS	38.9 L
NW	28.7 L
ZF	1.5 L
JF	6.5 L

Table S3. Variance inflation factors (VIFs) of environmental variables considered in the redundancy analysis (RDA). Variables with VIF > 10 were excluded from the final model.

Variable	VIF	Retained in final RDA
DO	2.14	Yes
COD	4.86	Yes

NH_4^+-N	5.73	Yes
NO_3^--N	2.91	Yes
TN	12.48	No
TP	10.67	No
PO_4^{3-}-P	4.22	Yes
Cu	2.76	Yes
Zn	3.18	Yes
Mn	2.43	Yes
